# Intersection of co‐occurring α‐synuclein and tau seeds in Lewy body and other neurodegenerative diseases

**DOI:** 10.1002/alz.090745

**Published:** 2025-01-09

**Authors:** Hannah Zamore, Matteo Manca, David G Coughlin, Heidi Standke, Danielle F. Browne, Denis Smirnov, Olivia R. Thomas, Caitlin Swanberg, Douglas R. Galasko, Annie Hiniker, Allison Kraus

**Affiliations:** ^1^ Case Western Reserve University, Cleveland, OH USA; ^2^ Department of Neurosciences, University of California San Diego, La Jolla, CA USA; ^3^ Department of Neurosciences, University of California San Diego, La Jolla, CA, San Diego, CA USA; ^4^ University of Southern California, Los Angeles, CA USA

## Abstract

**Background:**

Distinct amyloid structures characterize specific proteinopathies, including tau and α‐synuclein based neurodegenerative diseases. However, how protein seed co‐occurrence and other pathologic features account for clinicopathological heterogeneity observed within and between proteinopathies is unclear. Here, we quantify α‐synuclein and isoform‐specific tau seeds across neurodegenerative diseases, including in Lewy body disease (LBD), AD neuropathologic change (ADNC), and 4R tauopathy cases to inform how co‐occurring seeds may impact disease presentation and trajectory.

**Method:**

Real‐time quaking‐induced conversion (RT‐QuIC) assays with fM sensitivity were used to estimate α‐synuclein and tau isoform specific seeding doses in LBD (n=21), ADNC (n=47) across a range of Braak stages, and in 4R tauopathy (CBD=6; PSP=15) in mid‐frontal lobe and hippocampus.

**Result:**

α‐synuclein seeding doses were observed in the mid‐frontal lobe of all LBD cases, increasing with Lewy body stage (F=69.8, p<0.001) and reaching up to 10^5^ and 10^6^ seeding doses. Co‐occurring α‐synuclein seeding was noted as prevalent (50%) in AD, albeit typically at multi‐log lower levels than that quantified in LBD. 100% of AD with amygdala‐predominant α‐synuclein (N=4) and 30% of AD cases without Lewy bodies (N=10) had detectable α‐synuclein seeding. 3R/4R tau seeds were nearly ubiquitously detected well before overt neuropathology across all cases, and α‐synuclein seeding was also observed occasionally without α‐synuclein neuropathology. Markedly, 3R/4R tau and α‐synuclein seeding activities correlated in LBD while controlling for Braak stage (R^2^=0.72, p<0.001). 4R tau seeds confirmed 4R tauopathy and occurred to some extent, albeit with lower frequency than 3R/4R tau, in non‐4R tauopathy.

**Conclusion:**

Protein seed co‐occurrence is prevalent, including at early stages of both tau and α‐synuclein based disease. RT‐QuIC assays can selectively amplify, with multi‐log differentiation, α‐synuclein or tau seeds related to distinct α‐synuclein or tau structures characteristic of LBD, AD, and 4R tauopathies. Pervasive, but quantitatively distinct α‐synuclein seeding activity occurred in frontal cortex in LBD and AD, including detectable α‐synuclein seeds in some AD cases without identifiable Lewy body neuropathology. Regression analysis of seeding activities revealed quantitatively similar levels of α‐synuclein and tau seeding in LBD suggesting a possible novel role for α‐synuclein‐tau interaction in LBD, which we will explore in future studies.

